# Therapeutic potential of cyanobacteria against streptozotocin-induced diabetic rats

**DOI:** 10.1007/s13205-016-0411-0

**Published:** 2016-03-26

**Authors:** Muthuraman Pandurangan, Doo Hwan Kim

**Affiliations:** Department of Bioresources and Food Science, Konkuk University, Seoul, South Korea

**Keywords:** Streptozotocin, Cyanobacteria, Enzymes, Rats, Diabetes

## Abstract

Hypoglycemic effect of cyanobacteria has evaluated in the normal and streptozotocin-induced diabetic male albino rats as a mammalian model. Normal and streptozotocin-diabetic rats were orally administered cyanobacteria for 60 consecutive days, and their blood levels of glucose, insulin, C-peptide, lipid peroxidation, body weight and enzyme changes were determined using standard methods. Cyanobacteria administration reduced blood glucose level, and increased plasma insulin, C-peptide levels and restored the body weight. Cyanobacteria administration significantly reduced lipid peroxidation in the diabetic rats. Hexokinase enzyme activity was increased, whereas glucose-6-phosphatase enzyme activity decreased in streptozotocin-diabetic rats compared to their respective controls. Cyanobacteria administration caused significant renormalization of serum hepatic enzymes in streptozotocin-induced diabetic rats. In conclusion, cyanobacteria have a protective effect on anti-oxidant and anti-diabetic in streptozotocin-induced diabetic rats.

## Introduction

Diabetes mellitus is a metabolic disorder in which there are high blood glucose levels over a prolonged time. Type 1 diabetes mellitus is due to the body’s failure to produce enough amount of insulin. Type 2 diabetes mellitus starts with insulin resistance, a condition in which cells unable to respond to insulin properly (Muthuraman et al. 2014a, b). The number of diabetics is tremendously increasing in developing countries due to lifestyle (Sridhar et al. 2002). The careful control of blood glucose is a vital part of the management of diabetic complications (Muthuraman and Srikumar 2009). There are several anti-diabetic drugs available to control blood glucose level; however, these drugs produce side effects. There are five major kinds of anti-diabetic drugs such as sulfonylureas, meglitinides, biguanides, thiazolidinediones and alpha-glucosidase inhibitors available in the market. However, these drugs produce several side effects such as hypoglycemia, skin allergy, skin rash, gastrointestinal upset, fluid retention, edema, weight gain, lactic acidosis, nausea, diarrhea, liver function impairment and flatulence. Therefore, a search of the novel anti-diabetic drug without any side effect is mandatory in the clinical research.

The intravenous injection of streptozotocin in male albino Wistar rats makes pancreas swell and causes degeneration of Langerhans islet beta cells of the pancreas and induces experimental diabetes mellitus within 72 h. Streptozotocin induces one type of diabetes which is similar to diabetes mellitus with non-ketosis hyperglycemia. Induction of diabetes with Streptozotocin decreases nicotinamide adenine dinucleotide in pancreatic islet beta cells and causes histopathological effects in beta cells of the pancreas which probably intermediates induction of diabetes.

Several natural products have isolated from a broad range of taxa and tested for biomedical potential. These active components from plant origin have provided several crucial molecules for drug designing. The search of products from natural sources has revolutionized the programs of drug discovery. Several plant-derived molecules have shown a promising potential effect in therapeutics. The use of plant medicines in the treatment of several diseases such as central nervous system disorders is a traditional practice. The cyanobacteria (*spirulina platens*) have been recognized for their wide abundance. Cyanobacteria are considered as good candidates for applications in agriculture, food industry and pharmaceuticals. Antibacterial, antiviral, antifungal, algaecides and cytotoxic activities of cyanobacteria have been reported (Senthilkumar and Ahmed John 2008). Cyanobacteria contain secondary metabolites, bioactive peptides, macrolides and glycosides (Matthew et al. 2010). Cyanobacteria occur naturally in tropical and subtropical lakes with high pH and high concentrations; therefore, their concentrated nutritional profile makes it ideal for those preferring a whole-food supplement to artificial nutrient sources. A search for novel compounds is essential to conquering diabetes mellitus without side effect. Also, cyanobacterial strains have been well characterized, with some anti-oxidant and anti-inflammatory potential (Nagasathya and Thajuddin 2003; Rajavel et al. 2009). Our previous study evaluated the effect of cyanobacteria on plasma insulin and increased by regeneration of pancreatic beta cells in alloxan induced diabetic rats following administration (Muthuraman et al. 2009). The present study was therefore undertaken to investigate the chronic effect of cyanobacteria in streptozotocin-induced diabetes in male albino rats.

## Materials and methods

Male Wistar strain albino rats weighing 160–180 g have used for the investigation. The animals have housed under controlled temperature and hygiene conditions in propylene cages. Commercial rat chow with free access to drinking water ad libitum was provided for the rats. Experiments were carried out in accordance with internationally accepted ethical guidelines for the care of laboratory animals. A freshly prepared solution of streptozotocin (40 mg/kg) in 0.1 M citrate buffer was injected intraperitoneally (i.p.). Following 3 and 4 days of streptozotocin administration, blood was collected from the rats by eye bleeding method. Rats exhibiting a blood glucose level more than 300 mg/dl were believed to be diabetic and selected for the experiment. A total of 24 rats were used for the study. Rats were divided into four groups of six animals each: group 1: normal control rats, group 2: normal rats given cyanobacteria (20 mg/kg body weight), group 3: diabetic control rats, group 4: diabetic rats given cyanobacteria (20 mg/kg body weight). Cyanobacteria were administered orally using oral gavage for 60 consecutive days. Normal saline acted as a vehicle. At the end of 60 days, blood was collected from the animals by eye bleeding method for the further experiments.

### Preparation of the cyanobacteria extract

Cyanobacteria have cultivated by mass culture. Biomass was collected by centrifugation, washed twice with distilled water, and freeze-dried. The freeze-dried biomass was mixed with distilled water to give a 10 % (w/v) suspension and heated at 95 °C for 30 min. The extract was purified by centrifugation at 10,000 rev/min for 15 min and stored lyophilized until use (Muthuraman et al. 2009).

### Biochemical profile

Blood glucose in each sample was estimated by the standard method (Haidari et al. 2012). Plasma insulin was measured by radioimmunoassay method (Life Technologies, Korea) (Ezuruike and Prieto 2014). The C-peptide level was assayed by the chemiluminescence immunoassay method (Sigma-Aldrich, Korea) (Jones and Hattersley 2013). The body weight of rats was also determined.

### Enzymes

Glycolytic enzymes such as hexokinase (Muthuraman and Srikumar 2010) and glucose-6-phosphatase (Kirana and Srinivasan 2010) have measured by kit methods (Sigma-Aldrich, Korea). Serum hepatic marker enzymes such as alanine transaminase (ALT), aspartate transaminase (AST) and gamma-glutamyl transpeptidase (GGT) were measured by kit methods (Life Technologies, Korea) (Muthuviveganandavel et al. 2008).

### Lipid peroxidation

Lipid peroxidation (LPO) was determined by the kit method (Cell Biolabs, Inc, Korea). This was based on the spectrophotometric method of Muthuraman et al. (2014a, b). MDA was measured by determining the thiobarbituric acid reactive species. The absorbance of the resultant product has measured at 534 nm (Agilent Technologies, Cary 100 UV–Vis spectrophotometer).

### Statistical analysis

All values have expressed as mean ± SEM. Statistical analysis has carried with the use of SPSS 11 software. The statistical significance of differences between the means has assessed by student “*t*” test. A difference at *p* < 0.05 has considered statistically significant.

## Results

Cyanobacteria administration caused significant changes in the levels of glucose, plasma insulin, C-peptide, lipid peroxidation, body weights and serum hepatic marker enzymes in diabetic rats compared to control rats. Six rats were used for each analysis (*n* = 6). Glucose levels elevated and plasma insulin levels decreased significantly in streptozotocin-induced diabetic rats. Body weight also seemed to be increased compared to a normal rat. However, the chronic administration of cyanobacteria restored these parameters to normal levels.

### Blood glucose, insulin, and C-peptide

**Fig. 1:**
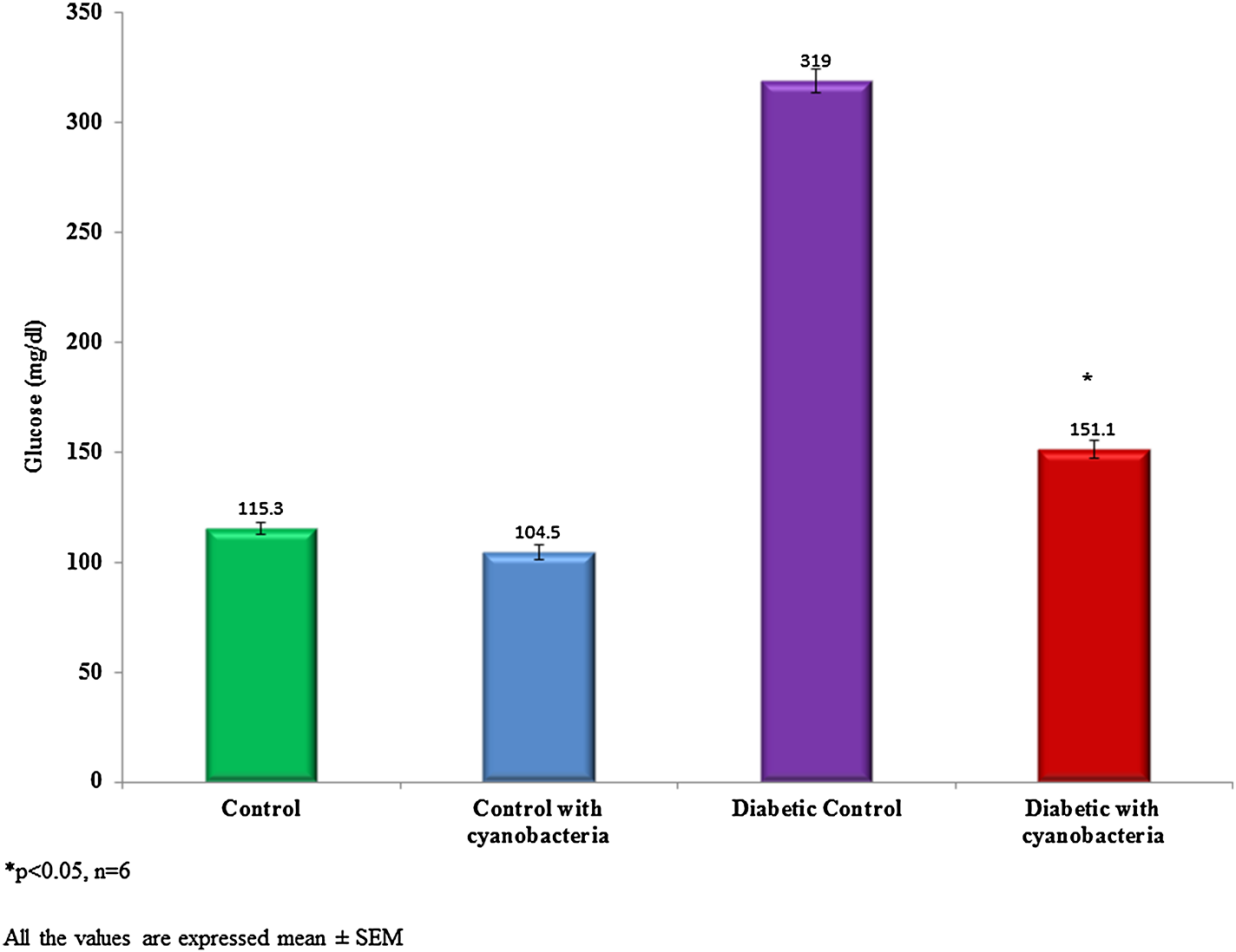
Effect of cyanobacteria on serum glucose level in normal and streptozotocin-induced diabetic male albino rats following 60 consecutive days of dose (20 mg/k.bwt) administration. All the values were expressed as mean ± SD, *p < 0.05, *n* = 6

**Fig. 2:**
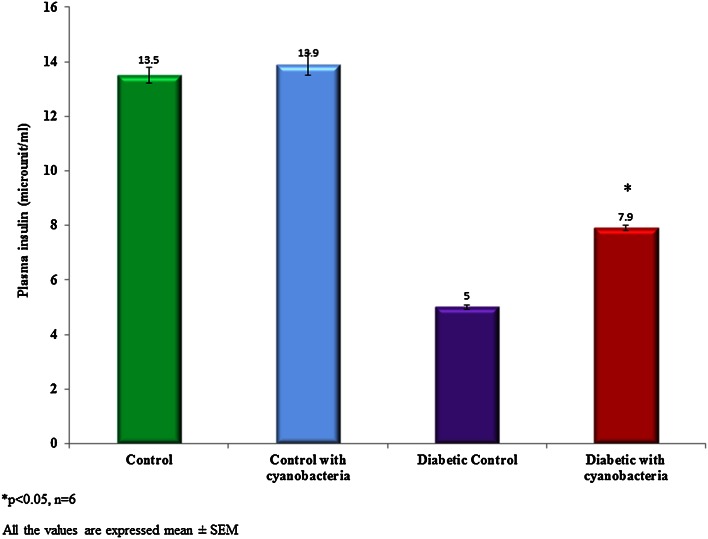
Effect of cyanobacteria on plasma insulin level in normal and streptozotocin-induced diabetic male albino rats following 60 consecutive days of dose (20 mg/k.bwt) administration. All the values were expressed as mean ± SD, *p < 0.05, *n* = 6

**Fig. 3:**
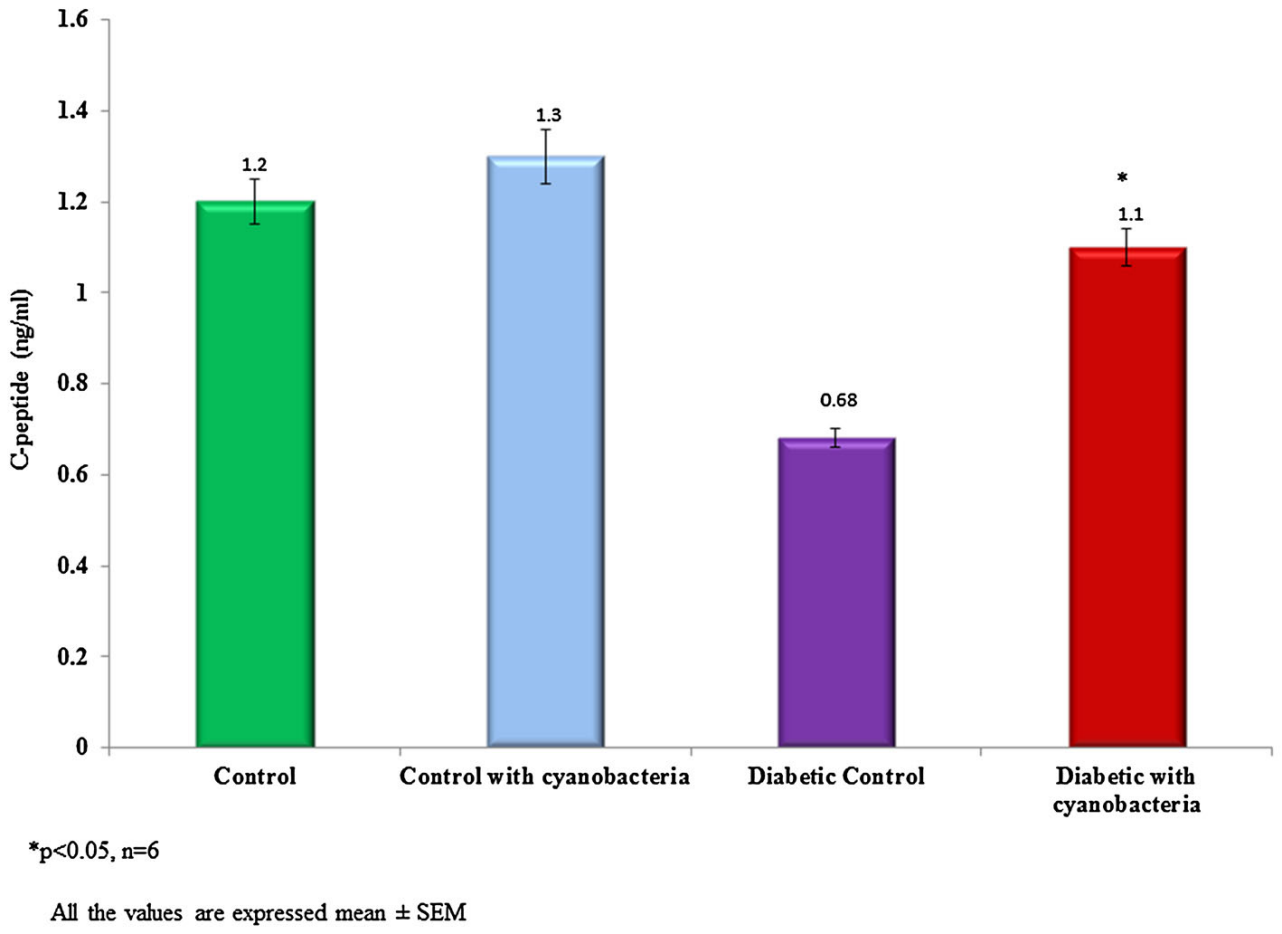
Effect of cyanobacteria on the C-peptide level in normal and streptozotocin-induced diabetic male albino rats following 60 consecutive days of dose (20 mg/k.bwt) administration. All the values were expressed as mean ± SD, *p < 0.05, *n* = 6

Blood glucose content was significantly reduced to 151.1 ± 4.1 mg/dl in streptozotocin-induced diabetic rats following cyanobacteria administration, whereas it was 115.3 ± 2.7 mg/dl. The blood glucose level of the diabetic rats selected for the study was above 300 mg/dl (Fig. 1). The plasma insulin level was found to be lower 5 ± 0.08 microunit/ml in the streptozotocin-induced diabetic rats when compared to normal rats (13.5 ± 0.3 microunit/ml). Cyanobacteria administration significantly increased the plasma insulin level 7.9 ± 0.1 microunit/ml in diabetic rats, and to a lesser degree in normal rats (Fig. 2). A C-peptide level also was found to be lower 0.68 ± 0.02 ng/ml in the streptozotocin-induced diabetic rats when compared to normal rats. Cyanobacteria administration significantly increased the C-peptide level 1.1 ± 0.04 ng/ml in the male albino diabetic rats (Fig. 3).

### Body weight changes

Cyanobacteria reversed weight loss in diabetic rats and yielded a lesser effect in the normal rats. At the end treatment time, body weights of rats were 195 ± 3.4, 188 ± 5, 140 ± 3 and 173 ± 4.1 g b.wt in group I, group II, group III and group IV, respectively. The body weight changes have calculated before and after cyanobacteria administration.

### Glycolytic enzyme activity

**Fig. 4:**
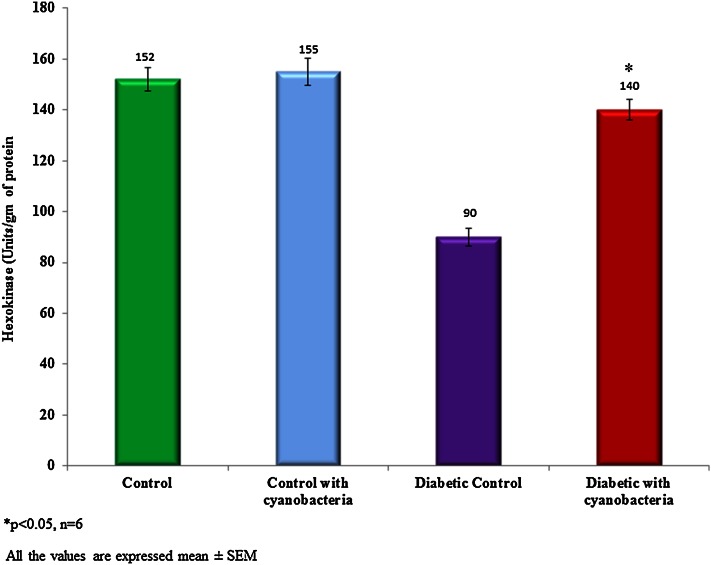
Effect of cyanobacteria on serum hexokinase enzyme activity in normal and streptozotocin-induced diabetic male albino rats following 60 consecutive days of dose (20 mg/k.bwt) administration. All the values were expressed as mean ± SD, *p < 0.05, *n* = 6

**Fig. 5:**
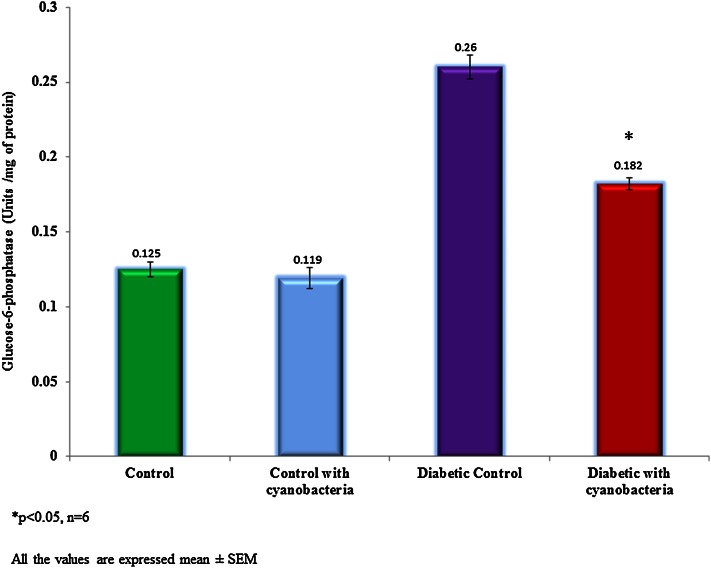
Effect of cyanobacteria on serum glucose-6-phosphatase activity in normal and streptozotocin-induced diabetic male albino rats following 60 consecutive days of dose (20 mg/k.bwt) administration. All the values were expressed as mean ± SD, *p < 0.05, *n* = 6

Cyanobacteria administration increased hexokinase activity 140 ± 4 units/mg of protein in streptozotocin-induced diabetic rats, whereas lesser (155 ± 5.3 units/mg of protein) extent in normal rats (Fig. 4). Cyanobacteria administration reduced glucose-6-phosphatase activity 0.182 ± 0.004 units/mg of protein in diabetic rats, whereas minor reductions in the normal rat (Fig. 5).

### Lipid peroxidation

**Fig. 6:**
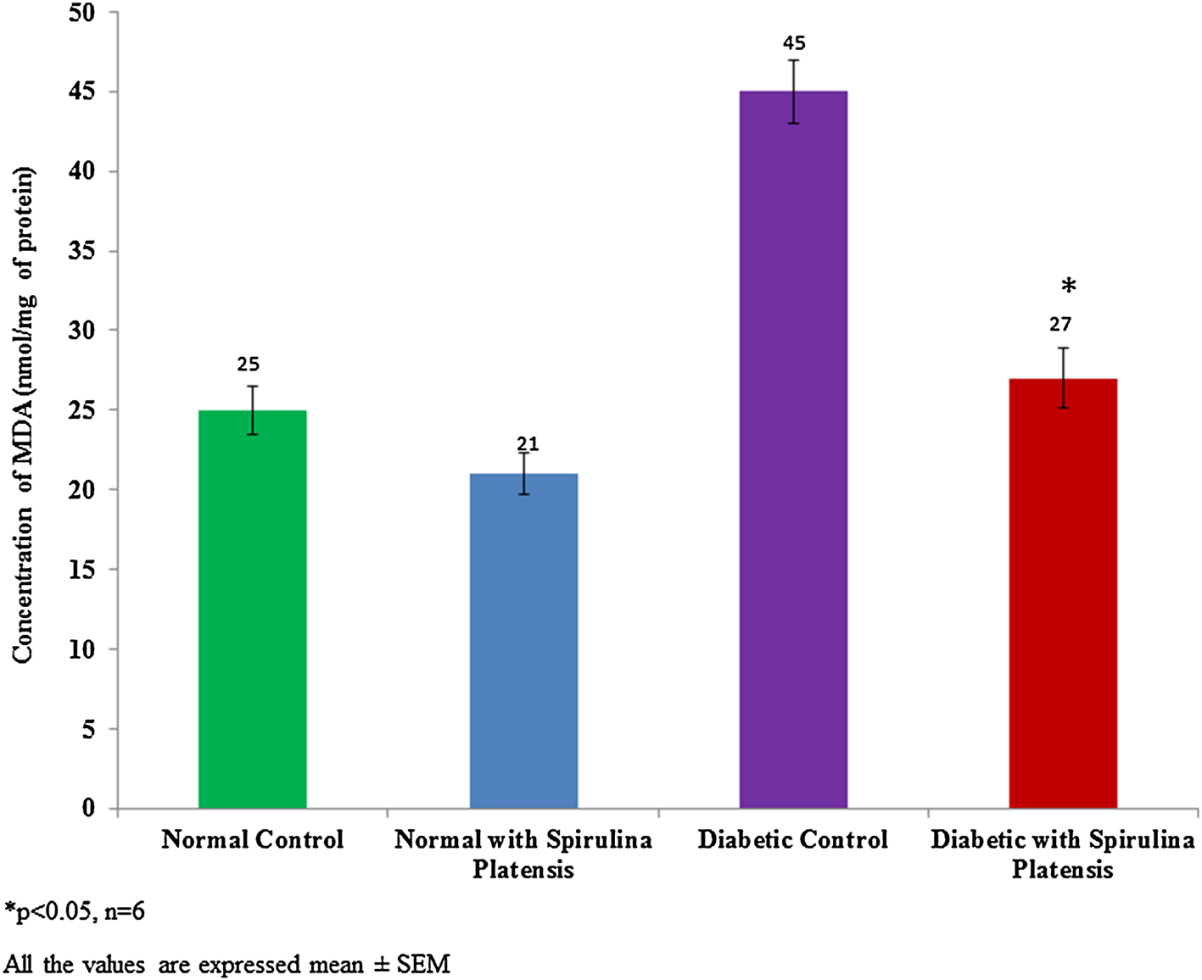
Effect of cyanobacteria on lipid peroxidation in normal and streptozotocin-induced diabetic male albino rats following 60 consecutive days of dose (20 mg/k.bwt) administration. All the values were expressed as mean ± SD, *p < 0.05, *n* = 6

The level of lipid peroxidation was found to be increased (45 ± 2 nmol/mg of protein) in streptozotocin-induced diabetic rats. Cyanobacteria administration significantly reduced lipid peroxidation level 27 ± 1.9 nmol/mg of protein in diabetic rats, whereas it was an insignificant reduction in normal rats (Fig. 6).

### Serum hepatic marker enzymes

The activity of serum hepatic marker enzymes such as ALT, AST, and GGT enzyme activity is elevated 42.0 ± 1.8, 92.3 ± 4.2 and 31.6 ± 1.5 U/L, respectively, in the streptozotocin-induced diabetic rats. Cyanobacteria administration significantly reduced these enzyme levels, whereas minor reductions in normal rats (Table 1).

**Table 1:** Effect of cyanobacteria on serum hepatic marker enzyme activity in normal and streptozotocin-induced diabetic male albino rats following 60 consecutive days of dose (20 mg/k.bwt) administration

Serum hepatic marker enzymes	Control	Control with cyanobacteria	Diabetic control	Diabetic with cyanobacteria
ALT (U/L)	21.9 ± 0.5	20.1 ± 1.2	42.0 ± 1.8	26.0 ± 1.3***
AST (U/L)	56.2 ± 2.1	54.0 ± 3.0	92.3 ± 4.2	66.5 ± 2.7*
GGT (U/L)	17.5 ± 0.9	15.7 ± 0.8	31.6 ± 1.5	20.1 ± 0.6*

All the values are expressed mean ± SEM

* *p* < 0.05; *** *p* < 0.001, *n* = 6

## Discussion

We evaluated the chronic effect of cyanobacteria on diabetic-biochemical markers such as blood glucose content, plasma insulin, C-peptide, lipid peroxidation, weight changes and glycolytic, serum hepatic marker enzyme changes in streptozotocin-induced diabetic male albino rats. Also, cyanobacteria were administered to the normal rats to evaluate the effect in normal condition. Streptozotocin has been widely used for diabetes induction in animal studies (Graham et al. 2011). Streptozotocin induced diabetes as indicated by the symptoms such as glycosuria, hyperglycemia, polyphagia, polydipsia and body weight loss (Deeds et al. 2011).

Cyanobacteria administration significantly renormalized altered biochemical markers in streptozotocin-induced diabetic rats. Glucose, plasma insulin, C-peptide, lipid peroxidation and enzyme levels have evaluated in the control and treated groups. Cyanobacteria administration significantly reduced the glucose level in the streptozotocin-induced diabetic rats, whereas no significant reduction in normal rats, which agrees with our previous findings (Muthuraman et al. 2009). Cyanobacteria administration increased plasma insulin and C-peptide levels in the diabetic rats to the near normal levels, while no significant changes have observed in the normal rats. Increased plasma insulin and C-peptide levels were indicative of the fact that cyanobacteria may enhance the secretion of insulin from the pancreas.

Also, cyanobacteria administration results in gaining weight in diabetic rats, and this may be due to its anti-hyperglycemic potential. Increased glucose-6-phosphatase could utilize for the conversion of carbohydrates to fat. Increased hexokinase enzyme activity indicates the higher rate glycolysis (Anitha and Chandra 2006). Hexokinase and glucose six phosphatase enzyme activities have not significantly changed in the normal rats following cyanobacteria administration. Reduced lipid peroxidation in diabetic rats indicates the anti-oxidant potential of cyanobacteria. ALT, AST, and GGT are indicators of hepatic damage (Whitehead et al. 1999). These enzymes increased in streptozotocin-induced diabetic rats due to hepatic damage. Cyanobacteria administration reduced the activities of these enzymes to the near normal levels. Therefore, the anti-hypoglycemic action of cyanobacteria mediated through potentiation of pancreatic secretion of insulin from the intact beta cells of the islets.

## Conclusion

Our experimental results indicated that the cyanobacteria administration renormalized the glucose, insulin, C-peptide, lipid peroxidation and glycolytic enzymes. Also, administration of cyanobacteria leads to regaining body weight in diabetic rats. Cyanobacteria administration reversed the hepatic damage by renormalizing of serum hepatic marker enzymes. Taking all these together, cyanobacteria show anti-hypoglycemic action through potentiation of pancreatic secretion of insulin from the intact beta cells of the islets.
